# Mechanisms Involved in the Promoting Activity of Fibroblasts in HTLV-1-Mediated Lymphomagenesis: Insights into the Plasticity of Lymphomatous Cells

**DOI:** 10.3390/ijms221910562

**Published:** 2021-09-29

**Authors:** Giulia Rigotto, Barbara Montini, Adriana Mattiolo, Nayana Lazzari, Maria Assunta Piano, Daniel Remondini, Sandra Marmiroli, Jessika Bertacchini, Luigi Chieco-Bianchi, Maria Luisa Calabrò

**Affiliations:** 1 Immunology and Molecular Oncology, Veneto Institute of Oncology IOV—IRCCS, 35128 Padua, Italy; giulia.rigotto@iov.veneto.it (G.R.); barbara.montini1979@gmail.com (B.M.); adriana.mattiolo86@gmail.com (A.M.); nayana.lazzari@iov.veneto.it (N.L.); mariaassunta.piano@iov.veneto.it (M.A.P.); 2 Department of Physics and Astronomy, University of Bologna, and Istituto Nazionale di Fisica Nucleare, INFN, 40127 Bologna, Italy; daniel.remondini@unibo.it; 3 Department of Biomedical, Metabolic and Neuronal Sciences, University of Modena and Reggio Emilia, 41125 Modena, Italy; sandra.marmiroli@unimore.it (S.M.); jbertacchini@unimore.it (J.B.); 4 Department of Surgery, Oncology and Gastroenterology, University of Padua, 35128 Padua, Italy; luigi.chiecobianchi@unipd.it

**Keywords:** lymphomagenesis, ATLL, HTLV-1, stemness, plasticity, cancer stem cells

## Abstract

Among the mechanisms leading to progression to Adult T-cell Leukaemia/Lymphoma in Human T-cell Leukaemia Virus type 1 (HTLV-1)-infected subjects, the contribution of stromal components remains poorly understood. To dissect the role of fibroblasts in HTLV-1-mediated lymphomagenesis, transcriptome studies, cytofluorimetric and qRT-PCR analyses of surface and intracellular markers linked to plasticity and stemness in coculture, and in vivo experiments were performed. A transcriptomic comparison between a more lymphomagenic (C91/III) and the parental (C91/PL) cell line evidenced hyperactivation of the PI3K/Akt pathway, confirmed by phospho-ELISA and 2-DE and WB analyses. C91/III cells also showed higher expression of mesenchymal and stemness genes. Short-term coculture with human foreskin fibroblasts (HFF) induced these features in C91/PL cells, and significantly increased not only the cancer stem cells (CSCs)-supporting CD10^+^GPR77^+^ HFF subpopulation, but also the percentage of ALDH1^bright^ C91/PL cells. A non-cytotoxic acetylsalicylic acid treatment decreased HFF-induced ALDH1^bright^ C91/PL cells, downregulated mesenchymal and stemness genes in cocultured cells, and delayed lymphoma growth in immunosuppressed mice, thus hindering the supportive activity of HFF on CSCs. These data suggest that crosstalk with HFF significantly intensifies the aggressiveness and plasticity of C91/PL cells, leading to the enrichment in lymphoma-initiating cells. Additional research is needed to better characterize these preliminary findings.

## 1. Introduction

Adult T-cell Leukaemia/Lymphoma (ATLL) is a mature T-cell neoplasm associated with Human T-cell Leukaemia Virus type 1 (HTLV-1) infection [1]. HTLV-1 infection is also associated with several inflammatory disorders, and the most frequent is HTLV-1-associated myelopathy/tropical spastic paraparesis (HAM/TSP) [2,3,4].

ATLL is sporadically diagnosed in North America and Europe, but it represents 25% of all peripheral T-cell lymphoma in Japan, one of the HTLV-1 endemic areas. ATLL presents with relevant clinical heterogeneity and, on the basis of the site of lymphomatous infiltration, and the presence and degree of leukaemia, hypercalcemia and lactate dehydrogenase levels, it was initially classified into four main clinical subtypes: smouldering, chronic, acute and lymphoma [5,6,7]. Aggressive variants include the acute and lymphoma types. Patients with these forms experience multidrug resistance of lymphomatous cells and a large tumour burden, with massive infiltration of several organs and tissues, including the gastrointestinal tract, lungs, and skin.

Although HTLV-1 immortalizes in vitro and in vivo mature T-cells, ATLL arises in only 2–5% of infected individuals decades after primary infection, indicating that a fully malignant phenotype is acquired through a multistep process. Indeed, progression to ATLL requires the cooperation of critical viral factors, Tax and HBZ, genetic and epigenetic alterations of host cells, deregulation of the immune system, and players of stromal microenvironment [3,8]. The role of stromal components in HTLV-1-mediated lymphomagenesis is still poorly understood. In a physiological context, a cooperative assemblage of immune and stromal cell types with structural and regulatory extracellular matrix (ECM) proteins provides a vigilant scaffold to normal cell functions. In lymphomagenesis, the dynamic crosstalk of haematopoietic and non-haematopoietic elements was recognized as essential for the pathogenetic processes that mediate the initiation and tissue-specific dissemination of distinct lymphoma subtypes [9,10]. Fibroblasts are one of the most abundant components in the stroma and a generous source of a variety of cytokines, growth factors and ECM proteins [11]. Their phenotypic and functional versatility gets a boost during tumorigenesis [12,13]. Indeed, cancer-associated fibroblasts (CAF) engage a dynamic and nuanced interaction with neoplastic cells and massively contribute to tumour cells heterogeneity [14,15]. A subset of CAF, defined by the co-expression of CD10 and GPR77 surface markers and identified in breast and lung tumours, was associated with cancer aggressiveness [16]. Specifically, this CAF subpopulation is linked to chemoresistance and constitutes a favourable niche for cancer stem cells (CSCs), thus promoting tumour development [16,17].

We previously demonstrated that fibroblasts are able to increase the oncogenic potential of C91/PL cells, a HTLV-1-immortalized human T-cell line. Indeed, when fibroblasts were co-injected with C91/PL cells into the peritoneal cavity of two different immunodeficient mouse strains, diffused lymphomatous masses developed at a higher frequency and/or with reduced latency, highlighting that the crosstalk with this cell type confers a growth advantage to C91/PL cells [18]. The C91/III cell line was derived through subsequent in vivo mouse passages of a lymphomatous mass, and was found to be able to induce aggressive lymphomas in NOD/SCID/IL2Rγ_c_ KO (NSG) mice, thus representing a new preclinical model mimicking the ATLL lymphoma variant [18]. Human foreskin fibroblasts (HFF) were shown to act through the induction of a secretory reprogramming in immortalized T-cells, sustaining the acquisition by premalignant cells of autonomous pro-lymphomagenic signals. The aim of the present study was to dissect the mechanisms involved in the pro-tumorigenic activity exerted by fibroblasts and leading to HTLV-1-mediated progression to overt lymphoma.

## 2. Results

### 2.1. C91/III Cells Display Transcriptional Reprogramming and Hyperactivation of the PI3K/Akt Signaling Pathway

**Figure 1:**
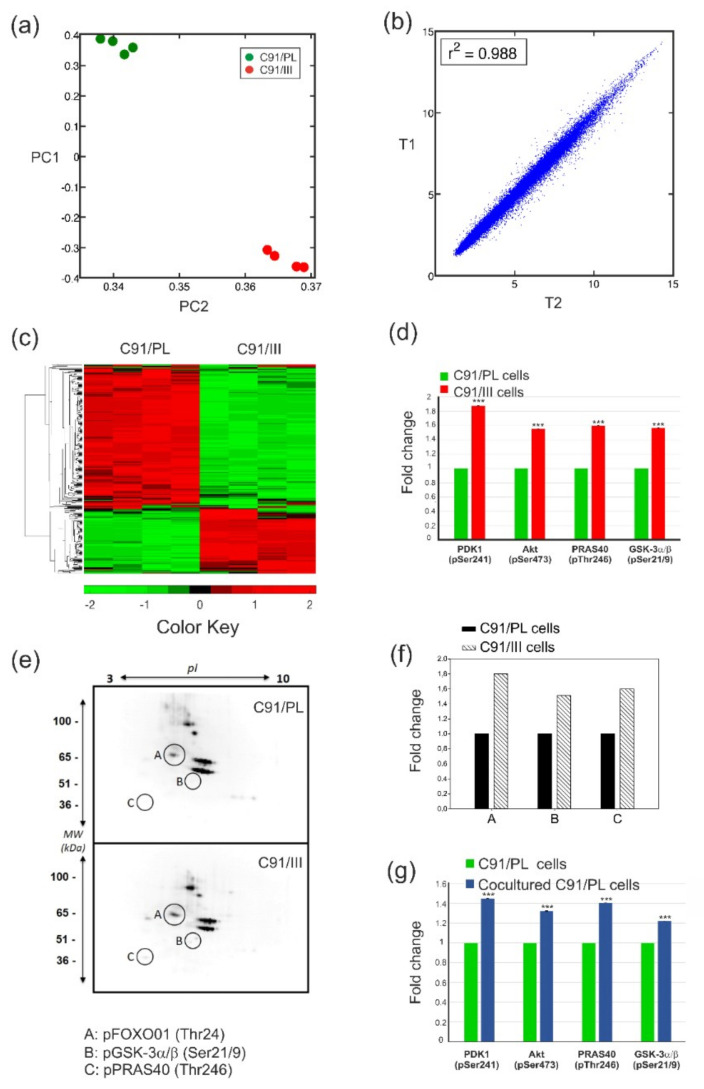
Analysis of transcriptional changes in C91/III cells and profiling of PI3K/Akt pathway activation in C91/III cells and HFF-cocultured C91/PL cells. (**a**) Whole-array principal component analysis (PCA), with a plot of the 1st and 2nd principal components (PC1 and PC2), showing a marked difference between the gene expression measurements of C91/PL (green) and C91/III (red) cells. (**b**) The comparison of C91/III cells pooled at two different time points (T1 and T2, one-month period), averaged over the two technical replicates, shows high similarity (Pearson’s correlation coefficient between the two profiles r = 0.994, r^2^ = 0.988), confirming the stability of the tumorigenic cell line over time. (**c**) A heatmap representing the bi-clustering of the 366 genes belonging to the KEGG PI3K/Akt signalling pathway. 105 genes were significantly modulated in C91/III cells (Q value < 0.0001 and *p* = 0.0000084). (**d**) PI3K/Akt pathway phosphorylation profiling. Phosphorylation of Akt and other key proteins of this pathway (PDK1, PRAS40 and GSK-3α/β) was higher in C91/III cells compared to C91/PL cells, indicating that the PI3K/Akt pathway was hyperactivated in the more lymphomagenic cell line. Data, obtained using phosphoprotein-specific ELISA assays, are shown as a ratio between the mean of the values measured in triplicate in C91/III cells and the mean of the values measured in C91/PL cells. The standard deviation (SD) of the ratio is calculated according to the theory of error propagation [19]. Statistical significance was determined by two-tailed Student’s *t*-test. *** indicates *p* < 0.001. (**e**) Two-dimensional electrophoresis (2-DE) combined with WB using an anti-Akt phospho-substrate antibody was carried out to confirm the hyperactivation of the PI3K/Akt pathway. Three substrates were found to be significantly more phosphorylated in C91/III (lower panel) compared to the parental cell line (upper panel), whose molecular weight and isoelectric point were found to match that of pFOXO01(Thr24), pGSK-3α/β(Ser21/9) and pPRAS40 (Thr246) (circled spots named A, B and C, respectively). (**f**) Representative histograms of the relative fold changes of the three circled phospho-substrates shown in panel **e**. (**g**) Phosphorylation of Akt and other crucial factors involved in the PI3K/Akt pathway was measured using phosphoprotein-specific ELISA assays in HFF-cocultured C91/PL cells, which showed a significant increase compared to C91/PL cells. Data were analysed and reported as described in panel **d**.

C91/III cells, derived from the C91/PL cell line, show the stable property of inducing aggressive T-cell lymphoma when intraperitoneally (i.p.) injected into immunodeficient mice [18]. A transcriptomic analysis of parental C91/PL vs. C91/III cells revealed that, among the 67,000 probes assayed, C91/III cells differentially expressed 5687 transcripts compared to C91/PL cells. Specifically, 3485 transcripts were significantly downregulated and 2202 significantly upregulated. The striking difference between the expression profiles of the two cell lines was evidenced by Principal Component Analysis (PCA) (Figure 1, panel a), whereas the linear distribution of the analysed parameters (panel b) demonstrated the stability of the results obtained with the two biological replicates of the two cell lines.

A functional enrichment analysis identified 15 biological pathways (*p* < 0.05, Table 1) differentially regulated in the two cell lines and relevant to ATLL pathogenesis. Notably, 105 out of the 366 genes involved in the PI3K/Akt pathway were significantly modulated (Figure 1, panel c). This signalling cascade is a master regulator of ATLL cell survival [20,21], and has been proposed as a therapeutic target in ATLL [22]. To confirm pathway activation, we analysed the phosphorylation of Akt itself, the Akt upstream regulator phosphoinositide-dependent kinase 1 (PDK1), and that of Akt direct substrates [proline-rich Akt substrate of 40 kDa (PRAS40) and glycogen synthase kinase 3alpha/beta (GSK-3α/β)], which have been demonstrated to be a very useful readout of signalling activity [23]. As shown in panel d, the phosphorylation of all the above proteins was significantly increased in C91/III cells compared to C91/PL cells, suggesting that this pathway is hyperactivated. Two-dimensional electrophoresis (2-DE) combined with WB using an anti-Akt phospho-substrate antibody was carried out to confirm this finding. Three substrates, pFOXO01(Thr24), pGSK-3α/β(Ser21/9) and pPRAS40(Thr246) were shown to be more phosphorylated in C91/III compared to the parental cell line (panels e and f), confirming the hyperactivation of the PI3K/Akt signalling pathway. Interestingly, C91/PL cells cocultured with HFF for 40 days showed the same pattern of increased phosphorylation (panel g), suggesting that the crosstalk with fibroblasts is able to hyperactivate this pathway.

### 2.2. Blockade of PI3K/Akt Signaling Pathway

**Figure 2:**
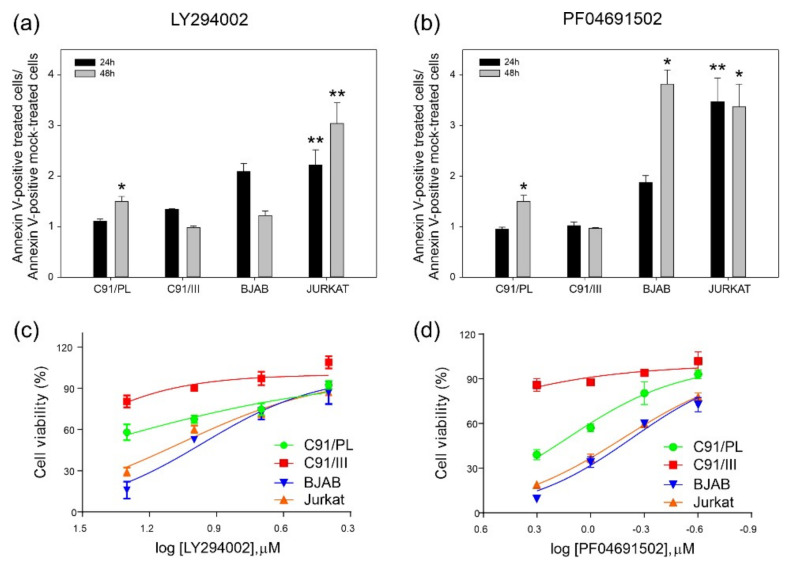
Analysis of the response of C91/PL and C91/III cells to blockade of the PI3K/Akt signalling pathway. C91/PL, C91/III, BJAB and Jurkat cells were treated with 10 µM LY294002 (**a**) or 1 µM PF04691502 (**b**) and apoptosis was evaluated 24 and 48 h after treatment. Data are reported as the ratio between the mean of the percentage of Annexin V–positive treated cells and mock-treated cells. Mock-treated cells were treated with corresponding dilutions of the diluent (dimethyl sulfoxide, DMSO). SD of the ratio was calculated as reported in Figure 1. Statistical significance was determined by two-tailed Student’s *t*-test. * indicates *p* < 0.05, and ** *p* < 0.01. Cell viability after 48 h of treatment was assessed by MTT assay and reported as dose–response curves of sensitivity to LY294002 (**c**) and PF04691502 (**d**) of C91/PL, C91/III, BJAB and Jurkat cells. Data (mean ± SD) are presented as the mean percentage of viable cells, where 100% viability was measured in cells exposed to complete medium only, calculated on two independent experiments performed in quadruplicate.

To investigate whether the upregulation of PI3K and downstream signalling described above rendered C91/III cells more sensitive to signalling blockade-induced cell death than parental cells, C91/PL and C91/III cells were cultured for up to 48 h in the presence of specific drugs, namely the PI3K inhibitor LY294002 or the dual PI3K/mTOR inhibitor PF04691502, and analysed after Annexin V staining. As a positive control, we used Jurkat and BJAB cell lines, known to be sensitive to these inhibitors. While some degree of apoptosis was triggered in all cell lines by a chronic blockade of PI3K signalling, although to a much greater extent in Jurkat and BJAB cells, contrary to expectations C91/III cells showed only a mild increase in positivity for Annexin V compared to C91/PL cells (Figure 2, panels a and b).

These results are in good agreement with the assessment of metabolic cell activity measured after 48 h by MTT assay (Figure 2, panels c and d). This assay confirmed that C91/III cells display very little response to PF04691502 (IC_50_ = 12.87 ± 0.3460 µM) even at very high concentrations, and no response at all to LY294002 (IC_50_ = 44.28 ± 0.1726 µM). Conversely, C91/PL cells were more sensitive to both drugs (IC_50_ LY294002 = 27.19 ± 0.07985 µM; IC_50_ PF04691502 = 1.354 ± 0.02488 µM), although to a much lower degree compared to Jurkat (IC_50_ LY294002 = 11.51 ± 0.02372 µM; IC_50_ PF04691502 = 0.6614 ± 0.01077 µM) and BJAB (IC_50_ LY294002 = 9.154 ± 0.02863 µM; IC_50_ PF04691502 = 0.5896 ± 0.02434 µM) cells, particularly at the highest concentration tested.

These findings indicate that, while both drugs were able to trigger massive apoptosis and impairment of cell metabolic activity in Jurkat and BJAB cell lines, hyperactivation of PI3K signalling rendered C91/III cells resistant, rather than more sensitive, to PI3K inhibition. This finding suggests that this pathway could not represent an actionable target for ATLL therapy in our model. 

### 2.3. In Vivo and In Vitro Effects of Short-Term Coculture of C91/PL Cells with HFF

**Figure 3:**
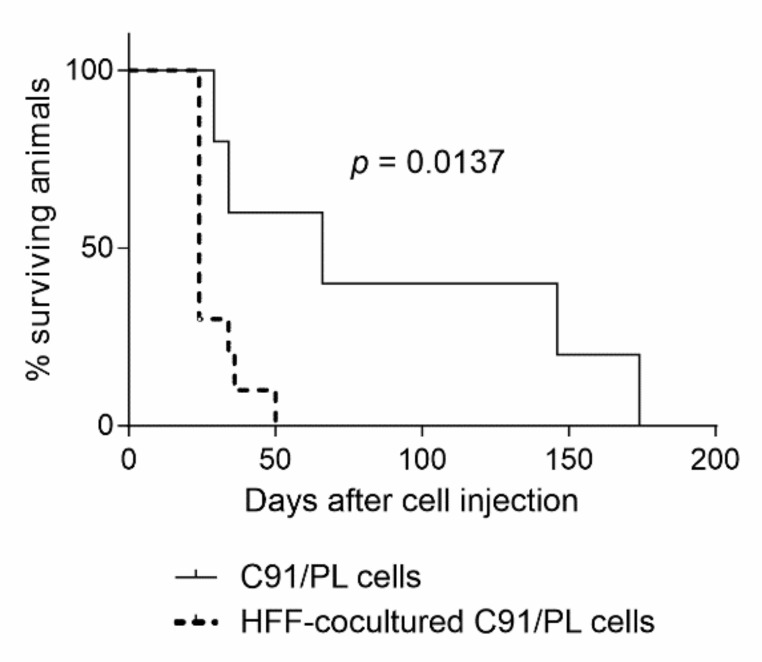
Tumorigenic capability of short-term HFF-cocultured C91/PL cells. Kaplan–Meier survival curves for 5-day-old NSG mice i.p. injected with 4 × 10^6^ C91/PL cells (5 mice) and with 4 × 10^6^ HFF-cocultured C91/PL cells (10 mice). A statistically significant reduction in the overall survival of mice injected with cocultured cells was observed, as reported. The log-rank test was used to evaluate the significance.

We previously showed that long-term HFF-coculture of C91/PL cells was able to reproduce many characteristics of the highly tumorigenic C91/III cells, including increased in vivo lymphomagenic activity [18]. We therefore analysed whether short-term coculture of C91/PL cells with fibroblasts might induce a more aggressive behaviour in vivo. As shown in Figure 3, C91/PL cells, cocultured with HFF for six days and i.p. injected into NSG mice, were able to induce aggressive lymphoma with a statistically significant (*p* = 0.0137) shorter latency compared to control cells.

**Figure 4:**
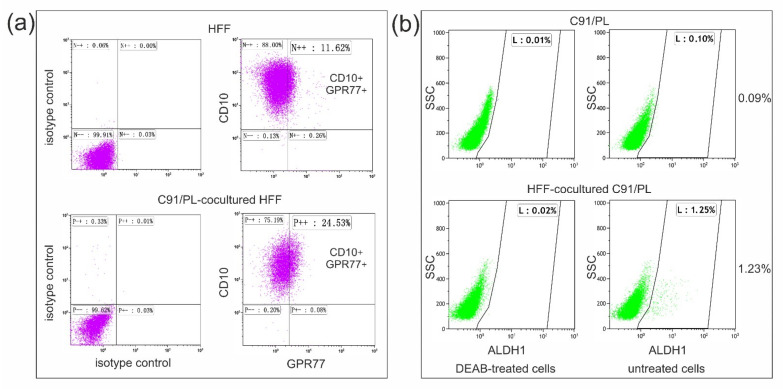
Characterization of HFF and C91/PL cells after short-term transwell coculture. (**a**) Flow cytometry analysis of CD10 and GPR77 expression in HFF in normal culture conditions (upper panels) and after 24 h of coculture with C91/PL cells (lower panels). Fibroblasts were stained with anti-CD10 and anti-GPR77 monoclonal antibodies; isotype controls (left-hand side panels) were used to determine background staining. A representative example of one of the four independent experiments is shown. The percentage of indicated CD10^+^GPR77^+^ HFF is enlarged. (**b**) Aldehyde dehydrogenase 1 (ALDH1) activity in C91/PL cells. Control C91/PL cells and HFF-cocultured C91/PL cells were collected at different time points and analysed for ALDH1 activity. Representative dot plots are shown for C91/PL cells alone (upper panels) or after 48 h of coculture with HFF (lower panels). N,N-diethylaminobenzaldehyde (DEAB) inhibitor was used to provide a negative control (left-hand side panels) for threshold set up. Flow cytometry data are shown as ALDH1 activity *vs* side scatter signal (SSC).

Short-term coculture was also used to dissect early changes involved in the intertypic crosstalk between fibroblasts and lymphomatous cells. The baseline phenotypic and secretory profiles of HFF used in these experiments have been previously reported [18]. As co-expression of CD10 and GPR77 surface markers was shown to identify a subset of CAF able to enhance cancer cell aggressiveness [16], we analysed the presence of this subpopulation in HFF in normal culture conditions and after coculture with C91/PL cells by cytofluorimetric analyses. Cocultures were carried out in transwell systems to avoid the possible interference of residual C91/PL cells, that were found to express GPR77 at high levels (median %: 85.4, range 76–93) (Figure S3, panel a). HFF co-expressed these markers in normal culture conditions at measurable levels (median %: 11.01, range 9.1–12.2) (Figure 4a). After 24–48 h of coculture with C91/PL cells, the percentage of CD10^+^GPR77^+^ HFF significantly increased (median %: 25.19, range 21.5–28.7, *p* = 0.00013, two-tailed Student’s *t*-test) (Figure 4a), suggesting that crosstalk with C91/PL cells significantly amplified this HFF subset. As this subpopulation of fibroblasts was shown to support CSCs in solid tumours, we studied markers linked to stemness in C91/PL cells at baseline and after coculture. Aldehyde dehydrogenase 1 (ALDH1) activity characterizes CSCs, as this enzyme is critical for the self-renewal of stem cells [24,25]. While C91/PL cells were almost devoid of ALDH1^bright^ cells (median %: 0.085, range 0–0.38), a small but significantly higher fraction of ALDH1^bright^ cells was found in C91/PL cells after 24–72 h of coculture with HFF (median %: 0.460, range 0.05–1.28, *p* = 0.0349, two-tailed Student’s *t*-test), as shown in Figure 4b. These experiments were carried out using transwell cocultures, as HFF show high basal ALDH1 activity (median %: 24.23, range 23.16–50.76) (Figure S3, panel b). Interestingly, ALDH1 activity was significantly higher (median %: 1.3, range 0.24–4.84; *p* = 0.0023, two-tailed Student’s *t*-test) in C91/III cells compared to the parental cell line (Figure S1), suggesting that their highly lymphomagenic activity might be associated with this subpopulation of lymphoma-initiating cells.

**Figure 5:**
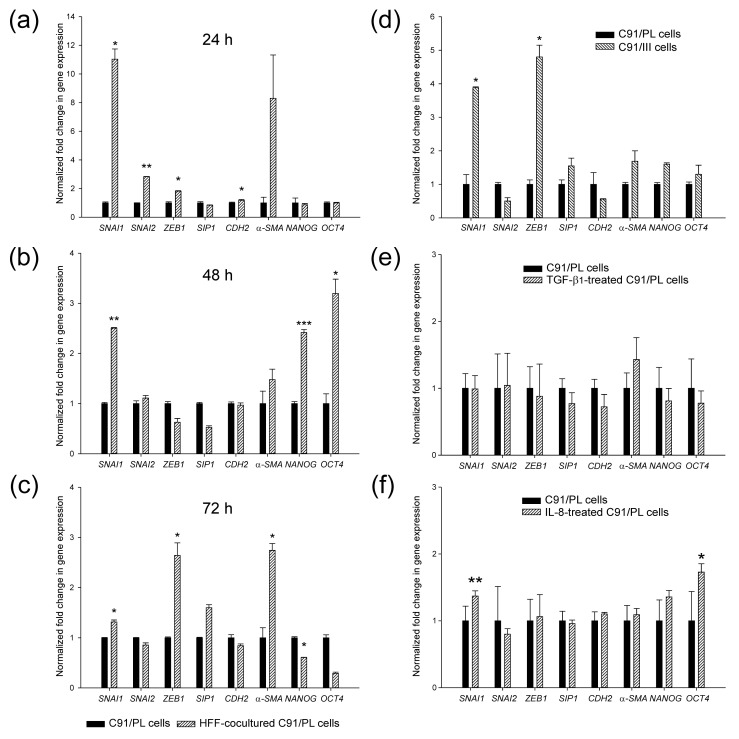
Expression of mesenchymal and stemness genes. Expression of mesenchymal (*SNAI1*, *SNAI2*, *ZEB1*, *SIP1*, *α-SMA* and *CDH2*) and stemness (*NANOG* and *OCT4*) genes were measured by quantitative real-time PCR in control and HFF-cocultured C91/PL cells collected after 24 h, 48 h and 72 h (**a**–**c**), in C91/III cells (**d**), and in C91/PL cells treated for 48 h with TGF-β_1_ (1 ng/mL) (**e**) and IL-8 (10 ng/mL) (**f**). Data (mean ± SD) are reported as fold change in gene expression using C91/PL cells as a reference. Statistical significance was determined by two-tailed Student’s *t*-test. * indicates *p* < 0.05, ** *p* < 0.01, and *** *p* < 0.001.

As tumour cells acquire stem cell properties through plasticity programs involving mesenchymal effectors [26], we analysed the induction of expression of mesenchymal and stemness markers in C91/PL cells after coculture by real-time qPCR. Cocultures were carried out using transwell systems to avoid the possible interference of residual HFF. As shown in Figure 5 (panels a–c), several mesenchymal markers were significantly upregulated after 24 h of coculture. *SNAI1*, *ZEB1* and *α-SMA* mRNAs were significantly overexpressed in cocultured cells at 72 h. The expression of *NANOG* and *OCT4*, two transcription factors required to maintain the pluripotency and self-renewal of CSCs, showed a significant upregulation after 48 h of coculture, supporting ALDH1 activity data, and their expression was downregulated at 72 h. Moreover, C91/III cells showed a statistically significant upregulation of *SNAI1* and *ZEB1* gene expression (panel d), suggesting that these mesenchymal effectors may be the ones more involved in the plasticity of these lymphoid cells. *ZEB1* was also found to be significantly upregulated in the transcriptomic analysis (as reported in S1_HTAresults.xls). To further analyse the soluble factors involved in C91/PL cell plasticity, we treated these cells with transforming growth factor beta_1_ (TGF-β_1_, panel e), a potent inducer of epithelial-mesenchymal transition (EMT), and with interleukin (IL)-8 (panel f), one of the factors secreted by HFF at high levels [18]. Of note, CD10^+^GPR77^+^ CAF were shown to induce a CSC increase through IL-8 secretion [16]. Only IL-8 induced a significant transcriptional upregulation of *SNAI1* and *OCT4* in C91/PL cells, thus indicating that this cytokine might contribute to CSC enrichment in our model of lymphomagenesis.

These data suggest that C91/PL cells engage a crosstalk with HFF, which increases their lymphomagenic activity through modulation of plasticity programs and by supporting their CSC subpopulation.

### 2.4. In Vitro and In Vivo Activity of Acetylsalicylic Acid (ASA, Aspirin) Treatment

**Figure 6:**
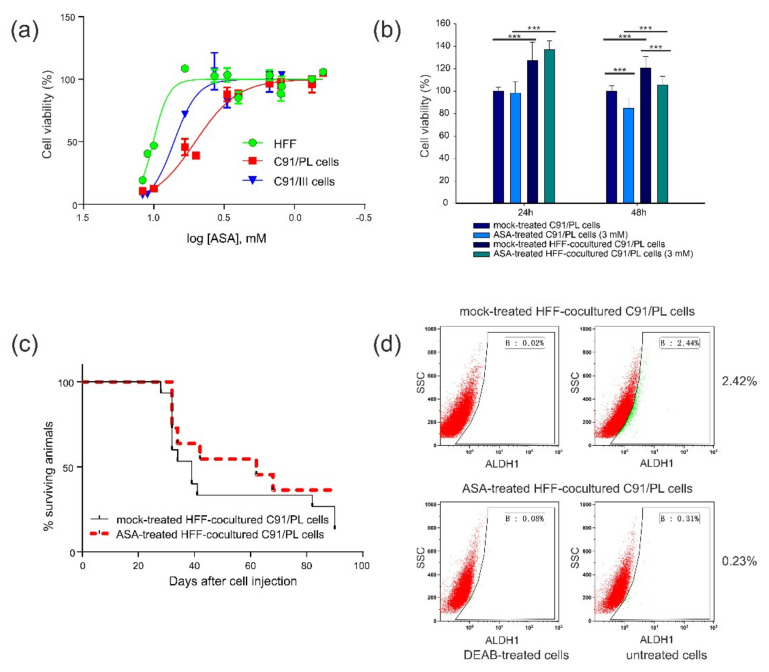
In vitro and in vivo activity of acetylsalicylic acid (ASA) treatment. (**a**) Dose–response curves of ASA sensitivity assayed in HFF, C91/PL and C91/III cell lines. Cell viability was analysed by MTT assay, which measures the metabolic activity of cells. The results are the mean and SD of three independent experiments performed in triplicate. (**b**) Viability of ASA-treated C91/PL cells. Viability, analysed by MTT assay at 24 h and 48 h, was evaluated in C91/PL cells exposed to 3 mM ASA in culture with or without confluent HFF. Data are reported as the ratio of the mean values obtained in ASA-treated, mock-treated HFF-cocultured and ASA-treated HFF-cocultured C91/PL cells/mock-treated C91/PL cells (%). SD of the ratio was calculated as reported in Figure 1. Results were obtained in two independent experiments performed in triplicate. Statistical significance was calculated by two-tailed Student’s *t*-test. *** indicates *p* < 0.001. (**c**) ASA anti-neoplastic activity in short-term HFF-cocultured C91/PL cells. Kaplan–Meier survival curves for six-day-old NSG mice i.p. injected with 4 × 10^6^ HFF-cocultured C91/PL cells in the presence (15 mice) or absence (12 mice, mock-treated with diluted DMSO) of 3 mM ASA for six days. (**d**) ALDH1 activity in C91/PL cells cocultured in transwell systems with HFF for six days and treated with 3 mM ASA or mock-treated with DMSO. Dot plots of C91/PL cells incubated with or without DEAB inhibitor and analysed for ALDH1 activity. DEAB was used to provide a negative control for threshold set up. Flow cytometry representative images are shown as ALDH1 activity vs. side scatter signal (SSC).

We subsequently analysed whether an anti-inflammatory drug, also known to interfere with stemness [27], might change the pro-tumorigenic activity exerted by HFF. We first measured the in vitro sensitivity of HFF, C91/PL and C91/III cells to ASA, a nonsteroidal anti-inflammatory drug (NSAID) known to exert antineoplastic activity in several in vitro and in vivo tumour models, not only through the attenuation of the pro-inflammatory response but also by decreasing the CSC subset [27]. As shown in Figure 6, panel a, C91/PL cells were found to be more sensitive to the cytotoxic activity of ASA (IC_50_ = 5.06 ± 1.03 mM) than C91/III cells (IC_50_ = 7.17 ± 1.05 mM) and HFF (IC_50_ = 10.08 ± 1.02 mM). The corresponding concentrations of the diluent (DMSO) did not significantly affect cell viability. In fact, the viability of DMSO-treated C91/PL cells was only marginally affected at the higher concentrations of the diluent (Figure S2), whereas DMSO-treated HFF were not affected. We therefore selected a non-tumoricidal dose of ASA, 3 mM, to evaluate its activity as a modulator of stemness and inflammation and assessed C91/PL cell viability in coculture at this concentration. As shown in Figure 6b, the higher metabolic activity in coculture was evident and statistically significant regardless of ASA treatment after 24 h of coculture. The viability of ASA-treated C91/PL cells decreased to 80% after 48 h, in accordance with the dose-response sensitivity assay (panel a); the same level of decrease was observed in coculture. 

To study the in vivo antineoplastic activity of 3 mM ASA, C91/PL cells were i.p. injected into six-day-old NSG mice after six days of coculture with HFF with ASA or mock treatment, as six days of coculture was shown to be sufficient to induce a more pathogenic phenotype in C91/PL cells, as shown above. As reported in Figure 6 (panel c), mice injected with ASA-treated HFF-cocultured C91/PL cells showed an increase in survival time (median overall survival, 62 days) compared to mice injected with mock-treated cocultured cells (median overall survival, 39 days), but the difference was not statistically significant. 

**Figure 7:**
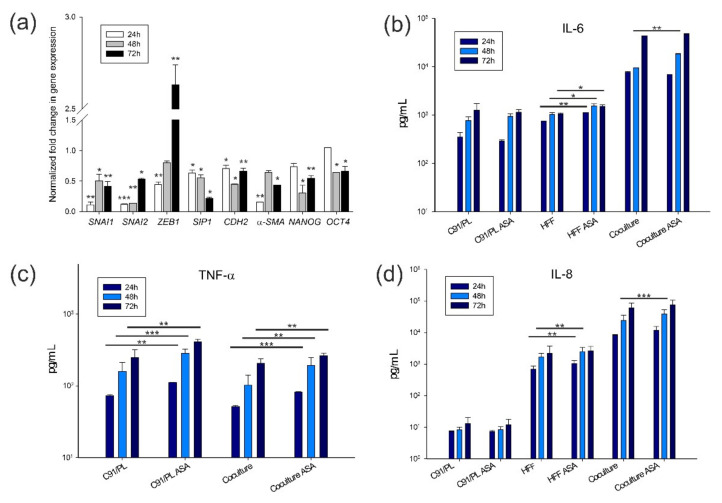
ASA activity on gene expression and release of inflammatory factors. (**a**) Modulation of mesenchymal and stemness genes in ASA-treated HFF-cocultured C91/PL cells. Fold change in gene expression was normalized on mock-treated HFF-cocultured C91/PL cells. Gene expression was measured in C91/PL cells cocultured in transwell systems. (**b**–**d**) IL-6, TNF-α and IL-8 released in culture supernatants by C91/PL cells, HFF and coculture after mock or 3 mM ASA treatment. HFF do not secrete TNF-α. Data are expressed in pg/mL. Statistical significance was calculated by two-tailed Student’s *t*-test. * indicates *p* < 0.05, ** *p* < 0.01, and *** *p* < 0.001.

ASA-treated cocultured cells were analysed for ALDH1 staining to study the effect of ASA on the CSC subpopulation of C91/PL cells. After 6 days of coculture, the percentage of ALDH1^bright^ cells was 2.43 ± 0.11%, whereas a significant decrease (0.22 ± 0.01%, *p* = 0.0210, two-tailed Student’s *t*-test) was observed in ASA-treated cocultured C91/PL cells (Figure 6d). This finding suggests that ASA treatment abrogates the supporting activity of HFF in CSC generation/maintenance. As ASA was found to decrease the percentage of ALDH1^bright^ cells induced by HFF, we studied the modulation of mesenchymal and stemness genes in the presence of ASA in HFF-cocultured C91/PL cells. As reported (Figure 7a), all genes, except for *ZEB1*, were significantly downmodulated in ASA-treated compared to mock-treated cocultured C91/PL cells. This suggests that ASA may decrease the expression of genes involved in tumour cell plasticity and stemness. *ZEB1* expression was only transiently downmodulated at 24 h, and showed a significant increase at 72 h. 

We also analysed the release of three proinflammatory factors, IL-6, tumour necrosis factor alpha (TNF-α) and IL-8, in the supernatants of C91/PL cells, HFF and cocultures as read-out of the possible anti-inflammatory activity of this concentration of NSAID. As shown in Figure 7 (panel b), ASA treatment did not significantly change the levels of IL-6 released by C91/PL cells, and significantly increased the amounts released by HFF at the three time points. The coculture showed a synergistic effect on the levels of the released cytokine, but 3 mM ASA treatment did not produce any modulatory effect, apart from that found at 48 h, with an increased IL-6 release in the coculture. Similarly, ASA treatment increased the release of TNF-α either in C91/PL cells alone or in coculture (Figure 7c). Released IL-8 showed a trend similar to that observed for IL-6 (Figure 7d). 

These preliminary findings indicate that ASA treatment, at a non-cytotoxic dose, does not exert a relevant anti-inflammatory activity but is able to abrogate the ability of HFF to modulate plasticity and stemness in C91/PL cells.

## 3. Discussion

The natural history of ATLL implies that several mechanisms contribute to the onset and progression of full-blown disease. Among these mechanisms, the biological principles of the crosstalk of pre-lymphomatous cells with microenvironmental non-immunological components have been poorly investigated and are largely underestimated as potential new therapeutic targets. In this study, we used C91/III cells as a model of HTLV-1-infected lymphomatous cells able to induce in vivo aggressive lymphomas [18]. Functional transcriptomic analysis revealed that many pathways involved in tumorigenesis were significantly modulated in C91/III cells (Table 1). We focused on the PI3K/Akt pathway, known to be critical to ATLL pathogenesis [20,21,22] since the expression of several genes involved in this pathway was significantly modified in C91/III cells and its functional engagement was confirmed by phosphorylation profiling (Figure 1). PI3K works as an extracellular sensor, and, through the binding of growth factors, activates Akt, the effector of the pathway [28,29]. The PI3K/Akt pathway exerts several pro-tumorigenic functions, through interaction with cellular proteins acting as cell cycle modulators, anti-apoptotic and transcriptional factors (TFs) [30]. We confirmed that Akt itself and several downstream effectors were indeed highly phosphorylated in C91/III cells (Figure 1), and that the HFF coculture was inducing the same profile of PI3K/Akt hyperactivation in the parental C91/PL cells, suggesting that crosstalk with the microenvironment can promote a pro-survival phenotype in lymphomatous cells. Enhanced PI3K/Akt activation was shown to be linked to mutant CCR4 in a fraction of ATLL samples and cell lines [31]. Sequence analyses of the carboxy-terminal region of CCR4 in C91/PL and C91/III cells did not reveal the presence of non-sense or frameshift mutations leading to a truncated chemokine receptor, suggesting that PI3K/Akt hyperactivation in C91/III cells was not due to gain-of-function mutations in CCR4 (data not shown). 

LY294002, like the majority of PI3K inhibitors, is a reversible ATP-competitive synthetic compound. Although its toxicity prevented clinical use, it is a very powerful tool in in vitro studies because of its specificity and efficacy. The antitumor activity of dual PI3K/mTOR inhibitors, such as PF04691502, as single agents and in combination with both solid cancers and in blood malignancy models is documented by a large body of preclinical data [32,33]. However, in contrast to what we observed in a model of primary effusion lymphoma [34] and acute myeloid leukaemia [35], the specific targeting of the PI3K/Akt signalling pathway did not induce substantial apoptosis in C91/III cells (Figure 2), suggesting that this pathway may not represent an actionable target for ATLL therapy in our model. This is in contrast with previous studies reporting the effect of PI3K signalling inhibition in ATLL. Specifically, two studies investigated the efficacy of idelalisib, a PI3K-δ isoform inhibitor, approved for chronic lymphocytic leukaemia (CLL) [22], and that of the dual, pan PI3K/mTOR inhibitor NVP-BEZ235 [36]. As only PI3K-δ isoform is expressed in CLL, idelalisib was initially found to be particularly effective in the treatment of this malignancy, but less active in other haematological disorders. Moreover, emergence of resistance to idelalisib was described also in CLL patients, in the absence of distinct resistance-associated mutations or deregulated signalling pathways. In other neoplasms, the specific inhibition of more than one PI3K isoform, as well as the dual inhibition of PI3K and mTOR, can increase the efficacy by affecting the survival of cancer cells both directly and through the action mediated by the microenvironment [37]. Indeed, these drugs might control the interactions of tumour and non-tumor cells leading to activation of PI3K and its effectors, such as the crosstalk triggered in the context of young cases of ATLL patients harbouring CTLA4-CD28 fusion [38]. The discrepancy between our results and the above studies may be explained by recent findings showing how cancer cells react to a signalling blockade. In fact, this highly active pro-survival pathway is sustained by considerable positive feedback [39], as also demonstrated by the failure of PI3K/Akt inhibitors as monotherapy in most clinical trials [40,41]. Thus, cancer cells adapt very rapidly to PI3K inhibition, mainly due to feedback signals leading to activation or enhanced expression of growth factor receptors, such as IR/IRS1/IGF-1R, which, in turn, reactivate PI3K [42,43].

Among the cell types populating the stromal microenvironment, fibroblasts contribute to several physiological and pathological mechanisms, revealing their extremely versatile nature [14]. Cancer-educated fibroblasts were shown to promote or restrain tumour progression through the secretion of soluble factors, recruitment of immunological regulators and effectors, ECM remodelling, direct contact with tumour cells and modulation of metabolism and angiogenesis [44]. Furthermore, HFF activity as a supporter of tumour-initiating cells or CSCs has been documented in solid tumours, whereas it has largely been neglected in lymphoid malignancies. A subset of fibroblasts, defined by the expression of specific surface markers, CD10 and GPR77, was shown to be highly represented in the microenvironment of chemo resistant lung and breast carcinomas [16]. Double-positive CD10^+^GPR77^+^ HFF were demonstrated to be involved in the generation of a niche favouring CSC survival [16]. Targeting these cells with antibodies against GPR77 reduced tumour formation and restored chemosensitivity in murine models of solid carcinomas [16]. Here, we show that coculture with C91/PL cells significantly increased the fraction of CD10^+^GPR77^+^ fibroblasts (Figure 4a), and this finding was paralleled by a significant increment in ALDH1^bright^ cells among HFF-cocultured C91/PL cells. Moreover, the crosstalk with fibroblasts was found to modulate the expression of stemness genes and mesenchymal TFs involved in tumour cell plasticity (Figure 5). Recently, it has been proposed that PI3K/Akt signalling might be involved in stemness enrichment in some cancer models, although the mechanisms remain completely unknown [45]. Therefore, increased stemness in C91/III cells and in cocultured C91/PL cells could be associated with hyperactivation of this pathway and further experiments are needed to prove this hypothesis. Interestingly, treatment with ASA, known to inhibit stemness properties in carcinoma cell lines and in preclinical models of solid tumours [27,46,47], was found to abrogate the increment of ALDH1^bright^ lymphomatous cells in coculture (Figure 6) and to downmodulate mesenchymal and stemness genes (Figure 7). Overall, these data suggest for the first time that a subset of HFF might support the subpopulation of lymphoma-initiating cells in our model of HTLV-1-induced lymphomagenesis. These data open up new perspectives for the therapy of aggressive ATLL subtypes through the targeting of a specific subset of fibroblasts in the tumour microenvironment, and disruption of the interplay between HFF and lymphomatous cells. 

The striking difference in the transcriptional program between the parental cell line and its more aggressive counterpart highlights the remarkable degree of plasticity of lymphomatous cells. Plasticity programs have been shown to be involved in solid tumour initiation and progression, and, more generally, imply dynamic changes between the epithelial and mesenchymal phenotypes with the appearance of intermediate/transitioning states, with different mixtures of epithelial and mesenchymal markers [48,49]. Indeed, in tumour cells, plasticity pathways modulate distinct biological properties linked to chemoresistance, stemness, migration, invasiveness and increased resistance to apoptosis. Apart from motility, which is an innate characteristic of components of the hematopoietic compartment, a worse clinical course and therapy resistance were linked to mesenchymal proteins in leukaemia and lymphomas, suggesting that these factors also modulate the aggressiveness in non-epithelial tumours [50]. These mesenchymal proteins are mainly TFs, which are emerging as relevant players in haematological disorders [51]. The major TFs are SNAIL, SLUG, ZEB1 and SIP1, and they control each other’s expression and cooperate with other TFs to regulate the expression of target genes. Whether they may exert an oncogenic function in an EMT-dependent or -independent manner has not been fully clarified in haematological disorders. It is conceivable that these TFs might modulate similar biological properties in different cell types, not necessarily in a context coupled with a canonical epithelial-mesenchymal axis. Although we have not performed functional analyses yet, our preliminary data indicate that C91/III cells are characterized by a higher expression of *SNAI1* and *ZEB1*, compared to the parental C91/PL cells (Figure 5). The upregulation of these mesenchymal TFs was concomitant with higher resistance to PI3K/Akt inhibitors and ASA, and an increment in the CSC subpopulation. Whether these features are directly linked to the higher expression of mesenchymal TFs remains to be proved.

The relevance of ZEB1 in ATLL pathogenesis is suggested by previous findings. Somatic mutations were found to affect *ZEB1* in ATLL, albeit at a low frequency [52]. Moreover, epigenomic and transcriptomic analyses of primary ATLL showed that *ZEB1* is epigenetically suppressed along with other TFs and zinc-finger proteins [53]. These studies also showed that ZEB1 is among the top five TFs acting on the promoter regions of epigenetically silenced miRNAs in ATLL [53], suggesting redundant silencing mechanisms, generally associated with disease progression. These findings are in line with its hypothetic role of oncosuppressor in ATLL pathogenesis, through enhancement of the TGF-β_1_-mediated growth inhibitory activity [54]. In fact, in vitro studies evidenced that *ZEB1* downmodulation, and the concomitant upregulation of the inhibitory SMAD7, could increase the resistance of ATLL cell lines to TGF-β_1_-mediated growth arrest [54]. These data support the hypothesis that intracellular EMT networks, such as those involving the SMAD signalling pathway, might modulate the plasticity of lymphomatous cells and govern some stages of HTLV-1-induced lymphomagenesis. In our model, TFs were upregulated during HFF coculture and *ZEB1* expression was not altered after treatment with exogenous TGF-β_1_ in C91/PL cells, suggesting that upregulation might have been triggered by other HFF-derived soluble factors. It is conceivable that interaction with the microenvironment may induce a modulation of EMT-TFs in our model, and this plastic/mesenchymal state, as with hybrid states in solid tumours, might represent an earlier stage compared to that found in full-blown lymphoma. 

Chronic inflammation by environmental pathogens, such as *Strongyloides stercoralis*, is often associated with HTLV-1 infection, and may trigger clonal proliferation of HTLV-1-infected cells with a higher proviral load, resulting in earlier onset of ATLL [55]. It has been demonstrated that cancer patients treated with aspirin show a reduced risk of recurrence and increased overall survival [56,57,58]. More recently, the regular use of low-dose aspirin was found to be associated with a decreased risk of non-Hodgkin’s lymphoma [59]. As cancer development is linked to chronic inflammation, the suppressive action of low-dose aspirin on this promoting state was evidenced in many in vitro and in vivo studies [60]. The anti-inflammatory activity of ASA is linked to the irreversible inhibition of cyclooxygenase 1 (COX-1) and COX-2 [61], with a high effect exerted on COX-1, a widely distributed and constitutive isoform, and, depending upon the dose, a lower efficacy on COX-2, the isoform expressed in sites of inflammation. Moreover, aspirin was shown to affect cancer stemness, as it was found to restore chemosensitivity in breast and colon carcinoma cells by decreasing the CSC subpopulation or by sensitizing CSCs toward chemotherapy [62,63]. Aspirin inhibits the pro-invasive phenotype of non-small cell lung carcinoma cell lines through *SNAI2* downregulation [64]. It was also shown to inhibit the proliferation, migration and TGF-β_1_-mediated EMT of the colorectal carcinoma (CRC) SW480 cell line [65]. ASA also counteracts lipopolysaccharides-induced EMT, migration and metastatic ability in murine models of C26 and HCT116 CRC cell lines [66]. In the present study, ASA was found to abrogate the increment of ALDH1^bright^ C91/PL cells triggered by the interplay with HFF. These findings corroborate previous evidence that aspirin interferes with the crosstalk, mediated by cytokines and angiogenic factors, between a breast cancer cell line and macrophages [67]. Furthermore, ASA treatment induced a significant decrease in the expression of mesenchymal EMT factors and transcriptional regulators of stem cell properties in lymphomatous cells (Figure 7), suggesting that a non-tumoricidal dose of aspirin may suppress the plasticity elicited by HFF in lymphomatous cells. However, the dose we used, administered to the coculture, was not sufficient to achieve a significant anti-neoplastic effect in vivo. This could be linked to the short-term treatment, limited to the coculture and not continued in vivo, and to the absence of a suppressive action on the release of the three inflammatory factors, IL-6, TNF-α and IL-8. IL-6 was shown to trigger spheroid formation in the MCF-7 breast cancer cell line, thus indicating that its pro-inflammatory loop mediates enrichment in mammary stem cells [68]. We observed that, while C91/PL cells did not change their release in this cytokine, HFF and the coculture showed a significant increase in IL-6 in the supernatants at 48 h, suggesting that this increment might have counterbalanced the initial delay in tumour development in vivo. This paradoxical exacerbation of the proinflammatory environment exerted by the NSAID treatment was observed in other experimental models and human disorders [69,70]. Therefore, in the context of aggressive ATLL forms, a low-dose aspirin might be used in combination with chemotherapy to lower the lymphoma stem cell subpopulation and prevent or reduce chemoresistance.

In conclusion, our preliminary data highlight that HFF may modulate the plasticity of HTLV-1-immortalized cells in our model, leading to the increased expression of mesenchymal and stemness genes and to a small subpopulation of putative lymphoma-initiating cells. These findings, which need to be extended by further in vitro and in vivo experiments, open up new perspectives on the prevention and treatment strategy of ATLL. 

## 4. Materials and Methods

### 4.1. Cell Lines, Cocultures and Mice

C91/PL and C91/III cell lines were obtained and cultured as previously described, as were primary HFF [18]. Specifically, C91/PL cells were originally obtained from Prof. Robin Weiss (Chester Beatty Laboratories, London). C91/PL and C91/III cells were authenticated by comparing the STR profile obtained with 18 genetic markers to that determined using the C91/PL cells received by the National Institute for Biological Standards and Controls (NIBSC), United Kingdom. HFF were a kind gift from Dr. Abatangelo and Dr. Zavan (University of Padova).

BJAB and Jurkat cells were obtained from DMSZ and ATCC, respectively, and were grown in RPMI 1640 (Merck, Darmstadt, Germany) supplemented with 10% foetal calf serum (FCS, Gibco, Thermo Fisher Scientific, Wilmington, DE, USA) and 2 mM L-glutamine (Gibco, Thermo Fisher Scientific). 

Long-term (40 days) cocultures of C91/PL cells with HFF were performed as previously described [18]. Short-term cocultures of C91/PL cells for in vivo experiments (6-days) were performed on semiconfluent HFF, seeded between the 12th and the 15th passage in T150 flasks, with the addition of fresh medium after 3 days. Short-term coculture experiments (24–72 h) for phenotypic, ALDH1, and apoptosis analyses, supernatant collection for ELISA assays, cell collection for mRNA quantification, and ASA treatment were carried out by plating 2 × 10^5^ HFF in T6-well plates. C91/PL cells were seeded at a density of 5 × 10^4^ cells/mL on the monolayer after 24 h. At least two technical and two biological replicates were made for each condition, in addition to cells alone as a control. As C91/PL cells tend to firmly adhere to HFF in direct coculture, all experiments in which a residual, albeit minimal, fraction of HFF or C91/PL cells might have interfered with the results obtained in the other cellular type were carried out using transwell systems. Cocultures in transwell systems were set up as previously reported [18], and control cells were seeded in the same culture conditions. ASA (Merck) was diluted in DMSO (2.5 M, stock solution) and tested in MTT assays using two-fold dilutions (from 12 to 0.750 mM and from 10 to 0.625 mM).

In vivo experiments were performed using 5/6-day-old non-obese diabetic/severe combined immunodeficient/IL-2 receptor common gamma chain KO (NOD/SCID/IL2Rγ_c_, NSG) mice (Charles River Laboratories, Calco, Italy). 

### 4.2. Transcriptomic and Bioinformatics Analyses

Global gene expression profile was analysed in C91/PL and C91/III cells using a high-resolution array that interrogates all transcript isoforms in the human transcriptome with about 67,500 probes targeting coding and non-coding transcripts (GeneChip Human Transcriptome Array 2.0 (HTA 2.0), Affymetrix eBioscience LTD, Hatfield, United Kingdom). The results reported in this study are mainly focused on coding transcripts, while those pertaining to non-coding transcripts will be presented elsewhere. Cellular pellets, prepared from each cell line in three different passages, were pooled and total RNA was extracted (TRIzol, Thermo Fisher Scientific). RNA concentration and purity were assessed using Nanodrop 1000 (Thermo Fisher Scientific). RNA integrity was evaluated using a 2100 Bioanalyzer (Agilent Technologies, Santa Clara, CA, USA). Two pools were conducted for each cell line and two technical replicates for each pool were analysed. Samples were analysed on different scales after GC-RMA processing (Affymetrix Power Tools software) and the evaluation of microarray boxplot uniformity. First, the Principal Component Analysis (PCA) was performed to highlight similarities between samples at whole-genome level. Second, single-gene analysis was performed between C91/PL and C91/III cell line samples by Student’s *t*-test, followed by Q-value post-hoc correction. Third, starting from a list of significant genes (Q < 10^−4^, corresponding to about 5700 genes) a functional enrichment analysis was performed considering the subset of 1340 significant genes mapped onto KEGG pathways (www.genome.jp/kegg, release 92.0, 1 Ocotober 2019) to find pathways with a significant number of differentially expressed genes (cumulative hypergeometric distribution test). 

### 4.3. Analysis of the PI3K/Akt Signaling Pathway

The activity of the PI3K/Akt signalling pathway was evaluated by specific phospho-ELISA kits and confirmed by 2-DE analysis combined with WB using an anti-Akt phospho-substrate (RXXS*/T*, Cell Signalling Technology, Danvers, MA, USA), as previously reported [71,72]. Synchronized cells were treated for 2 h with the PI3K inhibitor LY294002 (10 µM, Selleck Chemicals, Houston, TX, USA) or the dual PI3K/mTOR inhibitor PF04691502 (1 µM, Merck) and apoptosis was evaluated in C91/PL, C91/III, BJAB and Jurkat cells 24 and 48 h after treatment by flow cytometry after Annexin V/PI staining (Annexin-V-FLUOS Staining Kit, Merck). Two-fold dilutions of the two inhibitors were used to analyse the sensitivity of the four cell lines by MTT assay. Specifically, synchronized cells were seeded in 96-well plates (3 × 10^4^/well) and treated with LY294002 (from 2 μM to 0.25 μM) and PF04691502 (from 20 μM to 2.5 μM) for 48 h. IC_50_ was determined by a nonlinear regression analysis using the GraphPad Prism software (6.07 for Windows, San Diego, CA, USA). 

### 4.4. In vivo Experiments

To analyse the pro-tumorigenic activity of short-term HFF-coculture on C91/PL cells, ten five-day-old NSG mice were i.p. inoculated with 4 × 10^6^ C91/PL cells previously kept in coculture with HFF for six days. Five control five-day-old NSG mice were in parallel i.p. injected with 4 × 10^6^ C91/PL cells. The anti-neoplastic activity of 3 mM ASA was analysed by treating the coculture with ASA or with DMSO (mock-treated) and then using mock -treated and ASA-treated cocultured C91/PL cells as inoculum. Specifically, six-day-old NSG mice were i.p. injected with 4 × 10^6^ HFF-cocultured C91/PL cells in the presence (15 mice) of 3 mM ASA or diluent (12 mice) for six days. Mice were checked biweekly for cachexia and the presence of abdominal masses. For ethical reasons, tumour-bearing animals were culled when presenting signs of suffering and each mouse was considered dead from tumour progression on that date. At autopsy, the finding of organ involvement, and abdominal and pelvic tumour masses were considered as lymphoma growth. Fragments of infiltrated organs and tissues were fixed in 10% formalin and embedded in paraffin; sections were cut and haematoxylin–eosin staining was used for histological diagnosis.

### 4.5. Cytofluorimetric Analyses

HFF were analysed with the following anti-human monoclonal antibodies: allophycocyanin (APC)—conjugated mouse anti-human CD10 (clone eBioCB-CALLA, eBioscience, Thermo Fisher Scientific) in combination with phycoerythrin (PE)—conjugated mouse anti-human C5a receptor-like 2 (GPR77 or C5L2, clone 1D9-M12, BioLegend, San Diego, CA, USA) and, in parallel, with APC-IgG2b, k (eBioscience) and PE-IgG2a, k (BioLegend) isotype controls. To quantify ALDH1 activity, the ALDEFLUOR Kit (STEMCELL Technologies, Vancouver, BC, Canada) was used according to the manufacturer’s protocols. All analyses were performed on viable cells, stained with FVS780 dye (BD Biosciences, Milan, Italy).

Flow cytometry acquisition of stained cells was conducted using a LSR II flow cytometer (BD Biosciences) and data were analysed using Kaluza Analysis software Ver. 1.3 (Beckman Coulter, Brea, CA, USA).

### 4.6. Quantitative mRNA Analyses

Total RNA was extracted from cell pellets using the Quick-RNA™ Miniprep Kit (Zymo Research, Irvine, CA, USA) according to the manufacturer’s protocol. RNA concentration and purity were measured as described above. The expression of genes linked to a mesenchymal state (*SNAI1*, *SNAI2*, *ZEB1*, *SIP1*, *α-SMA* and *CDH2*/*N-cadherin*) and stemness (*NANOG* and *OCT4*) was assessed by quantitative real-time PCR (qRT-PCR), as previously reported [73]. Primer sets used in qRT-PCR were previously published [73,74,75]. The expression levels of analysed genes are reported as a relative quantification calculated using the human porphobilinogen deaminase gene as the endogenous control with the 2^−ΔΔCt^ method [76].

### 4.7. Quantitative Analyses of Soluble Factors

IL-6 and TNF-α were quantified in culture and coculture supernatants using ELISA assays (Human IL-6 Uncoated ELISA kit, Bender MedSystems, Vienna, Austria; Human TNF-α-ELISA Ready-SET-Go kit, Affymetrix eBioscience LTD, Hatfield, UK) according to the manufacturer’s instructions.

### 4.8. Statistical Analyses

Survival curves were estimated by the Kaplan–Meier method and compared with the log-rank test. Two-sided Student’s *t*-test was used to estimate statistical significance of different experimental conditions. *p*-values < 0.05 were considered significant.

## Figures and Tables

**Table 1:** Pathway enrichment analysis.

Pathway ID	Description ^1^	*p*-Value ^2^	N-Path ^3^	N-Sign ^4^
hsa04144	Endocytosis	1.55 × 10^−10^	255	92
hsa04512	ECM-receptor interaction	1.23 × 10^−8^	103	45
hsa04510	Focal adhesion	8.55 × 10^−8^	222	76
hsa04151	PI3K/Akt signalling pathway	6.73 × 10^−6^	366	105
hsa04020	Calcium signalling pathway	8.06 × 10^−5^	191	59
hsa03040	Spliceosome	0.000838	169	50
hsa05340	Primary immunodeficiency	0.00218	51	19
hsa04612	Antigen processing and presentation	0.00219	219	60
hsa04110	Cell cycle	0.00231	126	38
hsa04010	MAPK signalling pathway	0.0153	284	70
hsa04514	Cell adhesion molecules (CAMs)	0.0168	253	63
hsa04660	T cell receptor signalling pathway	0.0172	115	32
hsa05200	Pathways in cancer	0.0203	334	80
hsa04115	p53 signalling pathway	0.0483	67	19
hsa04370	VEGF signalling pathway	0.0483	67	19

^1^ Description of the Kyoto Encyclopaedia of Genes and Genomes (KEGG) pathway ID number; ^2^ *p*-values are in decreasing order of statistical significance; ^3^ Total number of genes in the array annotated in the KEGG pathway; ^4^ Number of differentially expressed genes between the C91/PL and C91/III cell lines.

## Data Availability

The results pertaining to transcriptomic data reported in this study are focused on coding transcripts. As results concerning non-coding transcripts will be presented elsewhere, transcriptomic data are available from the corresponding author upon reasonable request.

## Supplementary Materials

The following are available online at https://www.mdpi.com/article/10.3390/ijms221910562/s1, Figure S1: Aldehyde dehydrogenase 1 (ALDH1) activity in C91/III cells, Figure S2: Dose–response curves of sensitivity of C91/PL cells and HFF to 3 mM ASA and to diluent (DMSO, mock-treated). Figure S3. Characterization of HFF and C91/PL cells.

## Abbreviations

ALDH1	aldehyde dehydrogenase 1
APC	allophycocyanin
ASA	acetylsalicylic acid, aspirin
ATLL	Adult T-cell Leukaemia/Lymphoma
CAF	cancer-associated fibroblasts
COX-1	cyclooxygenase 1
COX-2	cyclooxygenase 2
CRC	colorectal carcinoma
CSCs	cancer stem cells
DEAB	N,N-diethylaminobenzaldehyde
DMSO	dimethyl sulfoxide
ECM	extracellular matrix
EMT	epithelial-mesenchymal transition
GSK-3α/β	glycogen synthase kinase 3
HFF	human foreskin fibroblasts
HTLV-1	Human T-cell Leukemia Virus type 1
IL	interleukin
i.p.	intraperitoneally
KEGG	Kyoto Encyclopedia of Genes and Genomes
NSAID	nonsteroidal anti-inflammatory drug
NSG	NOD/SCID/IL2Rγc KO
PCA	principal component analysis
PDK1	Akt upstream regulator phosphoinositide-dependent kinase 1
PE	phycoerythrin
PRAS40	proline-rich Akt substrate of 40 kDa
qRT-PCR	quantitative real-time PCR
SD	standard deviation
SSC	side scatter signal
TFs	transcription factors
TGF-β_1_	transforming growth factor beta1
TNF-α	tumor necrosis factor alpha
2-DE	two-dimensional electrophoresis
