# Supplementary material for: Mechanisms Involved in the Promoting Activity of Fibroblasts in HTLV-1-Mediated Lymphomagenesis: Insights into the Plasticity of Lymphomatous Cells
Source: Int J Mol Sci. 2021 Sep 29;22(19):10562. doi: 10.3390/ijms221910562 (PMC8508730; doi:10.3390/ijms221910562)
Supplement: Supplementary file 1 [file ijms-22-10562-s001.zip › ijms-1380660-supplementary.pdf]

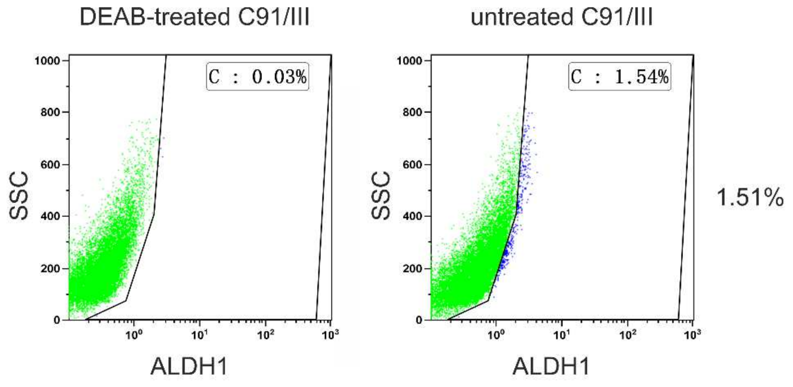

Figure S1. Aldehyde dehydrogenase 1 (ALDH1) activity in C91/III cells. Dot plots of C91/III cells incubated with or without N,N-diethylaminobenzaldehyde (DEAB) inhibitor and analyzed for ALDH1 activity. DEAB was used to provide a negative control for threshold set up. Flow cytometry representative images are shown as ALDH1 activity *vs* side scatter signal (SSC).

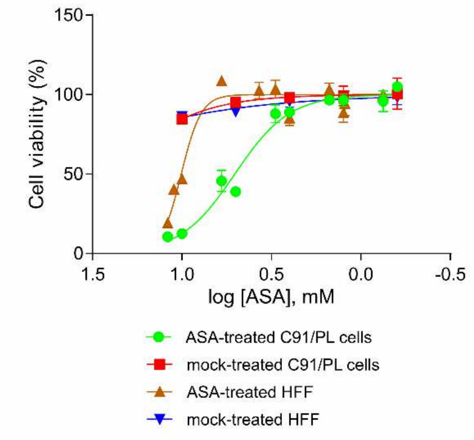

Figure S2. Dose–response curves of sensitivity of C91/PL cells and HFF to 3 mM acetylsalicylic acid (ASA) and to diluent (DMSO, mock-treated). The results are the mean and SD of three independent experiments performed in triplicate.

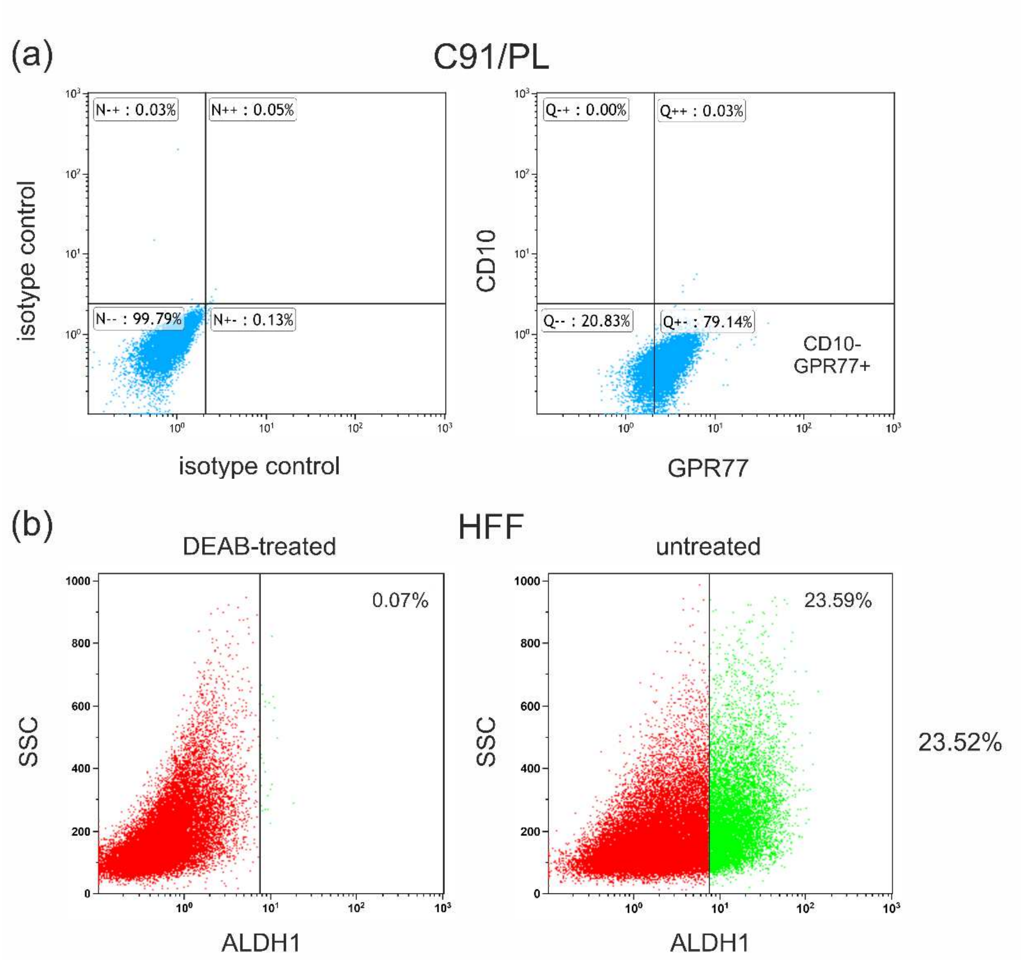

Figure S3. Characterization of C91/PL cells and HFF. (**a**) Flow cytometry analysis of CD10 and GPR77 expression in C91/PL cells. Cells were stained with anti-CD10 and anti-GPR77 monoclonal antibodies; isotype controls (left-hand side panel) were used to determine background staining. C91/PL cells do not express CD10 and the quadrant with $CD10^{-}GPR77^{+}$ cells is indicated. Representative dot plot images are shown. (**b**) ALDH1 activity in HFF. Dot plots of HFF incubated with or without DEAB inhibitor and analyzed for ALDH1 activity. DEAB was used to provide a negative control for threshold set up. Flow cytometry representative images are shown as ALDH1 *vs* SSC.
